# Efficacy of dietary supplements on sports performance outcomes: a systematic review of evidence in elite athletes

**DOI:** 10.3389/fnut.2025.1675654

**Published:** 2025-09-22

**Authors:** Tongwu Yu, Chuanwei Ding

**Affiliations:** ^1^Anhui Communications Vocational and Technical College, Hefei, China; ^2^Capital University of Physical Education and Sports, Beijing, China

**Keywords:** elite athletes, dietary supplements, ergogenic aids, sports performance, systematic review

## Abstract

**Objective:**

To systematically evaluate the efficacy of dietary supplements for enhancing athletic performance specifically in elite athletes, considering supplement type, dosing protocols, and sport-specific demands.

**Methods:**

This PRISMA-adherent systematic review (INPLASY202411036) searched PubMed, Web of Science, Scopus, and SportDiscus (Jan 2014-Nov 2024) for randomized controlled trials (RCTs) or controlled clinical trials examining dietary supplement interventions in elite athletes, compared to placebo/no intervention, reporting quantitative performance outcomes. Methodological quality was assessed using the PEDro scale; risk of bias was evaluated with the Cochrane Risk of Bias Tool V.2. Narrative synthesis was performed due to outcome heterogeneity.

**Results:**

Forty-six studies (*n* = 928 participants) met inclusion criteria, with predominantly male participants (60%). Performance enhancers showed varying efficacy: caffeine (3–6 mg/kg) consistently improved power output and technical performance; beta-alanine demonstrated sport-specific benefits; while nitrate supplementation showed limited effects in elite populations. Recovery supplements displayed mixed results, with amino acids and probiotics showing promise for fatigue prevention and exercise tolerance. Studies demonstrated high methodological quality (average PEDro score: 10.65/11), though female athletes were underrepresented (10% of studies).

**Conclusion:**

Dietary supplement efficacy in elite athletes is highly variable, contingent upon supplement type, sport-specific demands, individualized dosing protocols, and athlete characteristics (including potential gender differences). Caffeine and certain amino acid/probiotic formulations demonstrate the most consistent benefits. Findings strongly support individualized, evidence-based supplementation strategies over generic protocols. Future research must address the significant gender gap and underrepresentation of specific sports.

## 1 Introduction

The world of competitive sports is characterized by the ever-constant need to push the boundaries of sports excellence. Athletes constantly seek that extra edge whether a fraction of improvement that could mean the difference between them standing on the podium and watching from the sidelines. Premised on this quest and the overarching need to be an exceptional athlete, dietary supplements have become a constant companion for many athletes, particularly those competing at elite levels with usage stemming from local gyms to Olympic arenas. One could argue that dietary supplementation and related products have become as commonplace as water bottles and training logs. According to Maughan et al. (1), the use of dietary supplements is prevalent among elite athletes with many using to not only to enhance their performance but also to maintain their health and support recovery. A dietary supplement is defined as a commercially available product consumed in addition to the usual diet, including vitamins, minerals, herbs, amino acids, caffeine, and various other substances intended to supplement nutritional intake (2). The global market for dietary supplements over the years has registered tremendous growth with sales for sports nutrition supplements estimated to be US$5.67 billion in 2016 accounting for about 13.8% pf the US$41.6 billion market for dietary supplements are related products (3). This is a significant growth following the global market for support nutrition supplements was valued at US$4.2 billion in 2009 (4) while the dietary supplements market was expected to exceed US$46 billion in 2022 (5) with new projections showing the market is likely to exceed US$163.1 billion by 2016 (6). The statistics denote the expanding role and dependability of these products in both athletic and general populations.

The use of dietary supplements for sport performance enhancement is a common practice in the health and fitness arena with its use stemming from recreational and amateur to professional athletes indiscriminate of demographics. Current evidence suggests usage of dietary supplements among athletes typically ranges from 40% to 100%, with variability noted based on the type of sport, training intensity, competition level, age, and gender (7, 8). Kaufman et al. (9) note that 40-100% of trained athletes use supplements, often without proper guidance or evidence-based information. Peeling et al. (10) attribute the high usage of supplements among elite athletes compared to on-elite counterparts to the pursuit of marginal performance gains in highly competitive environments. This heightened prevalence among elite athletes reflects the intense pressure to maximize performance anchored on the belief supplementation provides a competitive edge.

Performance is not the only reason for the use of supplements for athletes cite a myriad of motivations, including but not limited, to recovery optimization, health maintenance, and injury prevention (11). Despite varying levels of scientific evidence supporting the efficacy of supplement usage, the perceived benefits have led to the widespread adoption across various sporting disciplines. For instance, Kaufman et al. (9) draw us to the use of some supplements such as caffeine, creatine, and beta-alanine that have substantial evidence supporting their performance-enhancing effects, while others lack robust scientific validation; however, all have varying implications that require personalized supplementation strategies and protocols.

Nonetheless, the widespread use of supplements among athletes raises several concerns. One of the primary contentious besides the unclear efficacy of supplements, is the unregulated nature of the supplementation industry compared to pharmaceutical products which leads to potential issues with product quality, safety, and labeling accuracy (1, 6, 7). Secondly, there is a risk of inadvertent doping due to contaminated supplements and inaccurate dosages or false labeling among others which potentially would result in anti-doping rule violations and severe consequences for athletes' careers and even the general health of the athletes (7, 8). These risks are particularly pertinent for elite athletes subject to regular anti-doping testing.

It is vital to note that the use of supplementation is a multiparty decision-making stemming from individual athletes, coaches, medical staff, and nutritionists. Moradi et al. (2) posit that supplementation decisions are frequently anchored on coaches are the primary source of information even though they may not always possess up-to-date information regarding the efficacy, accuracy, safety, quality and potential side effects. This highlights the importance of evidence-based decision-making and proper education regarding supplement use in sports. A systematic review by Knapik et al. (12) on the prevalence of supplement use found significant heterogeneity in supplement use patterns across different sports and athletic levels. The research further found higher supplement use rates among elite athletes, with some studies reporting over 90% use in certain sports. However, the methodological quality of many studies was generally low, with only 34% achieving half the available points on quality assessment scales.

Recent research has also identified differences in supplement use between male and female athletes, with variations in both the types of supplements used and the reasons for their use. Moradi et al. (2) reported female athletes are more drawn to iron supplements, while male athletes tend to use protein and creatine supplements more. These gender-specific patterns reflect different physiological needs and performance goals between male and female athletes. Given the high prevalence of supplement use, particularly among elite athletes, and the potential risks associated with their consumption, there is a critical need for a comprehensive understanding of current supplement use patterns, evidence of efficacy, and safety considerations. Therefore. We aimed through this systematic review to examine the evidence supporting the efficacy of commonly used supplements.

A comprehensive understanding of the efficacy of dietary supplements among elite athletes more so on their performance is imperative for the development of evidence-based guidelines for supplement use in sports. This would not only inform on the supplement and athlete safety but also optimize high sport performance enhancement. This review will provide valuable insights for athletes, coaches, sports medicine practitioners, and researchers in making informed decisions about supplement use in competitive sports. The primary aim of this systematic review was to evaluate the effectiveness of dietary supplements on performance enhancement in elite athletes. Through comprehensive analysis of randomized controlled trials, we examined how different supplements influence athletic performance across major sporting categories including endurance, combat, team, and individual sports. We investigated the impact of various dosing protocols and administration strategies to understand how timing, quantity, and duration of supplementation affect performance outcomes. Additionally, we sought to identify which supplements demonstrate consistent benefits for specific performance aspects such as power, endurance, and recovery, regardless of the sporting discipline. This approach allows us to provide evidence-based insights into supplement efficacy while considering both sport-specific demands and general performance enhancement principles. The findings offer practical guidance for athletes, coaches, and sports nutrition practitioners in making informed decisions about supplement use in competitive sports.

## 2 Methods

### 2.1 Protocol and registration

This systematic review protocol was registered with the International Platform of Registered Systematic Review and Meta-Analysis Protocols (INPLASY) on November 7, 2024 (registration number INPLASY202411036) (13). The protocol adhered to the Preferred Reporting Items for Systematic Reviews and Meta-Analyses (PRISMA) guidelines. The protocol was revised after registration to reduce the number of databases from 5 to four, data range from starting in 2004 to 2014 and change the title from focusing on competitive athletes to elite athletes as well as refine the search strategy as well as modified the quality assessment by replacing GRADE with PEDRO scale for quality assessment. The review is not financially supported by any organization or sponsor, and the authors declare no conflicts of interest.

### 2.2 Search strategy

**Figure 1:**
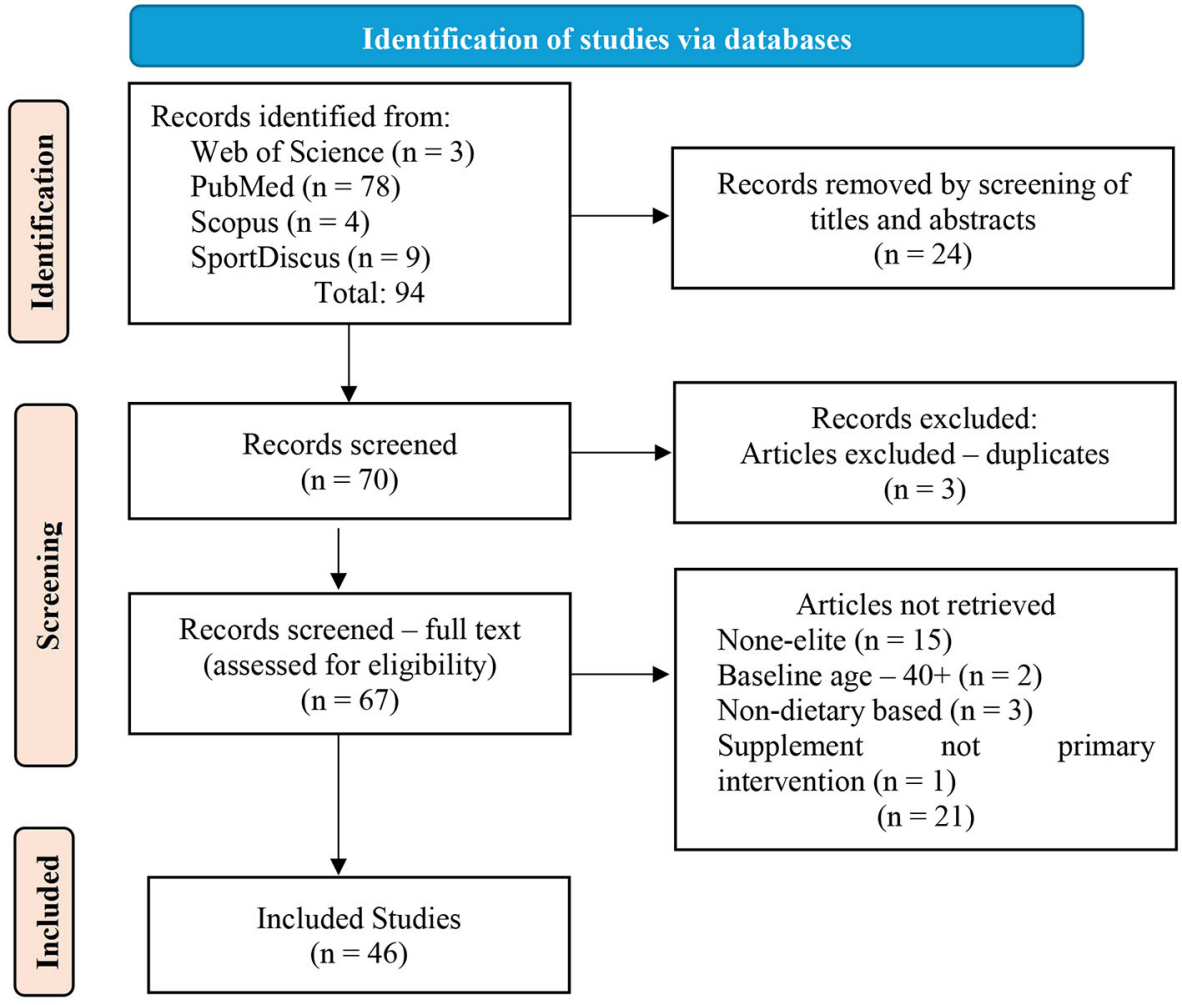
PRISMA flow chart for the included articles.

A comprehensive systematic search was conducted across four major electronic databases: PubMed, Web of Science, Scopus, and SportDiscus. The search period encompassed publications from January 2014 to November 2024. To ensure systematic rigor, we developed a structured search strategy using a combination of controlled vocabulary (MeSH terms) and free-text terms across three key concept areas. Population terms included “Athletes”, “elite athlete”, “professional athlete”, “Olympic athlete”, “world-class athlete”, “international athlete”, “high-performance athlete”, “elite sport”, “professional sport”, “Olympic sport”, and “national team”. The iIntervention terms were “Dietary Supplements”, “Ergogenic Acids”, “dietary supplement”, “ergogenic aid”, “supplemental nutrition”, “nutritional supplement”, “performance supplement”, and “sports supplement”. The outcome terms included “Athletic Performance”, “Physical Endurance”, “performance”, “power output”, “endurance performance”, “peak power”, “sports performance”, “competition performance”, and “elite performance”. The search strings were adapted for each database's specific syntax while maintaining consistent search concepts. Database-specific controlled vocabulary was incorporated where available to enhance search sensitivity. The complete search strategy for each database is detailed in Appendix 1. All search results were crosschecked for the removal of duplicates; the results of each database are recorded in Figure 1.

### 2.3 Eligibility criteria

#### 2.3.1 Inclusion criteria

Studies were included if they met all of the following criteria: (1) randomized controlled trials (RCTs) or controlled clinical trials including crossover studies; (2) healthy competitive athletes; (3) Any supplement that can be categorized as a dietary supplement for performance enhancement (e.g., beta-alanine, branched-chain amino acids (BCAAs), caffeine, creatine, dietary nitrates, multivitamins, protein supplements, vitamin D); (4) studies including comparison to no intervention or placebo; (5) studies reporting at least one measure of athletic performance; (6) articles published from 2014 to date (October 31, 2024) (10 years) and (7) studies involving only competitive athletes at elite levels.

### 2.4 Exclusion criteria

Studies were excluded if they met any of the following criteria: (1) non-English language publications; (2) studies on injured athletes; (3) studies without a control group; (4) non-human studies and (5) studies not reporting quantitative performance outcomes. Only studies published in English were included due to resource limitations for accurate translation. Only peer-reviewed publications were included. Conference abstracts, unpublished trials, and gray literature were excluded.

These criteria were applied independently by two reviewers during both the initial screening of titles and abstracts and the subsequent full-text review phase. Any disagreements were resolved by scoring the article against the inclusion and exclusion criteria. All inclusion and exclusion decisions were documented using a standardized form to ensure consistency and transparency in the selection process.

### 2.5 Data extraction and synthesis

We implemented a three-tier screening process to ensure systematic and unbiased study selection. The initial screening was completed by two independent reviewers who screened titles and abstracts against predetermined inclusion/exclusion criteria. Disagreements were resolved through discussion with a third reviewer. This was followed by full-text review where selected articles underwent full-text review by two independent reviewers using a standardized assessment form. Final selection required consensus between reviewers with disagreements resolved through structured discussion.

The data extraction process was conducted using a standardized form. The following information about study characteristics was extracted from each included study: authors and year of publication, study design and sport/activity name. Population demographics data extracted included the number of participants, sample size for each study group, gender distribution (number of males and females), and overall/group mean age (with standard deviation). The following supplement information was extracted supplement category (recovery, performance, ergogenic acids) and type (e.g. caffeine etc), manufacturer/supplier details when available, dosage and administration protocols and duration and frequency of supplementation. The following outcome measures were collected primarily focusing on primary performance outcomes and physiological markers. Key findings of the included articles were also assessed.

Given the variability in outcomes being reported across studies and the lack of studies measuring the same outcomes with the same methods, a meta-analysis was not undertaken to calculate pooled effect sizes on sports nutrition knowledge, attitudes, and practices of gym users. Therefore, a narrative synthesis approach was adopted. In studies where multiple time points were reported, data from all-time points were extracted to capture both immediate and sustained effects of supplementation. When studies reported multiple outcomes, all relevant outcomes meeting our inclusion criteria were extracted and included in the synthesis. The extracted data were organized by supplement type to facilitate comparison across studies and to identify patterns in the effectiveness of different supplements for specific performance outcomes. This organization also allowed for the identification of gaps in the current evidence base.

## 3 Quality assessment

The methodological quality of the included studies was assessed using the PEDro scale, which evaluates the internal validity and interpretability of randomized trials (14, 15). The risk of bias was assessed using the Cochrane Risk of Bias Tool V.2, examining (16–18): selection bias; performance bias, detection bias, attrition bias, deporting bias and other sources of bias.

## 4 Results

A comprehensive systematic search was conducted across four major electronic databases: PubMed (*n* = 78), Web of Science (*n* = 3), Scopus (*n* = 4), and SportDiscus (*n* = 9) (see Figure 1). The initial search yielded a total of 94 potentially relevant articles. The search strategy employed specific combinations of keywords and controlled vocabulary terms related to dietary supplements, athletic performance, and elite athletes. The initial screening process comprised 94 records which were screened based on their titles and abstracts resulting in the exclusion of 24 records whereas 3 more were excluded due to duplicates resulting in a total of 67 articles subject to the full-text review. The full-text articles were assessed for eligibility (*n* = 67), with exclusions based on the criteria noted in Figure 1 which led to the exclusion of 21 records. Forty-six studies (19–64) meet all inclusion criteria hence forming the final dataset for the systematic review. The screening and selection process was conducted systematically following the PRISMA guidelines to ensure thoroughness and transparency in the review process. Each stage of the selection process was documented, and reasons for exclusion were recorded to maintain a clear audit trail.

### 4.1 Risk of bias and methodological quality

The studies demonstrated strong attention to methodological quality through rigorous randomization procedures, implementation of appropriate blinding protocols, clear inclusion and exclusion criteria, standardized performance measures and controlled testing conditions. The PEDro scale assessing the methodological quality of articles revealed exceptionally high standards of research design and implementation. The average PEDro score was 10.65 out of 11, demonstrating robust methodological rigor across the reviewed studies (see Table 1). Notably, 39 studies (84.8%) achieved the maximum score of 11 points, indicating adherence to all quality criteria, while six studies (8.7%) scored 9 points (27, 28, 32, 36, 44, 62), and only one study (2.2%) scored 7 points (25). The majority of studies demonstrated exemplary compliance with fundamental methodological requirements as all 46 studies (100%) satisfied the criteria for eligibility criteria specification, blind assessors, between-group comparisons, point measures and variability reporting. Almost perfect compliance (98%) was observed in random allocation, baseline comparability, and blinding of both participants and therapists. The high adherence to these methodological standards significantly strengthens the reliability and validity of the research findings. However, some minor variations in quality were observed in areas of adequate follow-up and intention-to-treat analysis, where 42 (28, 32, 36, 44) out of 46 studies (91%) met these criteria.

**Table 1:** Methodological quality assessment of teh articles using PEDro scale.

**Study**	**EC**	**RA**	**CA**	**BC**	**BP**	**BT**	**BA**	**AFU**	**ITT**	**BGC**	**PMV**	**Total score**
Acar et al., 2024	1	1	1	1	1	1	1	1	1	1	1	11
Ávila-Gandía et al., 2021	1	1	1	1	1	1	1	1	1	1	1	11
Brisola et al., 2017	1	1	1	1	1	1	1	1	1	1	1	11
Broelz et al., 2018	1	1	1	1	1	1	1	1	1	1	1	11
Buzdagli et al., 2023	1	1	1	1	1	1	1	1	1	1	1	11
Camerino et al., 2016	1	1	1	1	1	1	1	1	1	1	1	11
Carvalho-Peixoto et al., 2015	1	0	1	0	0	0	1	1	1	1	1	7
Chen et al., 2016	1	1	1	1	1	1	1	1	1	1	1	11
Delleli et al., 2024	1	1	1	1	1	1	1	0	0	1	1	9
Durkalec-Michalski et al., 2017	1	1	1	1	1	1	1	0	0	1	1	9
Durkalec-Michalski et al., 2018	1	1	1	1	1	1	1	1	1	1	1	11
Durkalec–Michalski et al., 2020	1	1	1	1	1	1	1	1	1	1	1	11
Durkalec-Michalski et al., 2024	1	1	1	1	1	1	1	1	1	1	1	11
Evans et al., 2018	1	1	1	1	1	1	1	0	0	1	1	9
Fairbairn et al., 2018	1	1	1	1	1	1	1	1	1	1	1	11
Fernández et al., 2021	1	1	1	1	1	1	1	1	1	1	1	11
Filip-Stachnik et al., 2021	1	1	1	1	1	1	1	1	1	1	1	11
Fye et al., 2021	1	1	1	1	1	1	1	1	1	1	1	11
Gambardella et al., 2021	1	1	1	1	1	0	0	1	1	1	1	9
Garnacho-Castaño et al., 2018	1	1	1	1	1	1	1	1	1	1	1	11
Graham-Paulson et al., 2016	1	1	1	1	1	1	1	1	1	1	1	11
Guo and Wang, 2024	1	1	1	1	1	1	1	1	1	1	1	11
Gurton et al., 2024	1	1	1	1	1	1	1	1	1	1	1	11
Jodra et al., 2020	1	1	1	1	1	1	1	1	1	1	1	11
Jonvik et al., 2018	1	1	1	1	1	1	1	1	1	1	1	11
Karayigit et al., 2020	1	1	1	1	1	1	1	1	1	1	1	11
Kopec et al., 2016	1	1	1	1	1	1	1	1	1	1	1	11
Lara et al., 2015	1	1	1	1	1	1	1	0	0	1	1	9
Lewis et al., 2015	1	1	1	1	1	1	1	1	1	1	1	11
Liu et al., 2024	1	1	1	1	1	1	1	1	1	1	1	11
McQuillan et al., 2017	1	1	1	1	1	1	1	1	1	1	1	11
Mielgo-Ayuso et al., 2018	1	1	1	1	1	1	1	1	1	1	1	11
Miraftabi et al., 2021	1	1	1	1	1	1	1	1	1	1	1	11
Nyakayiru et al., 2017	1	1	1	1	1	1	1	1	1	1	1	11
Ojeda et al., 2023	1	1	1	1	1	1	1	1	1	1	1	11
Orlando et al., 2018	1	1	1	1	1	1	1	1	1	1	1	11
Paryab et al., 2021	1	1	1	1	1	1	1	1	1	1	1	11
Portillo et al., 2017	1	1	1	1	1	1	1	1	1	1	1	11
Przewłócka et al., 2023	1	1	1	1	1	1	1	1	1	1	1	11
Puente et al., 2017	1	1	1	1	1	1	1	1	1	1	1	11
Ramos-Campo et al., 2019	1	1	1	1	1	1	1	1	1	1	1	11
Schreiber et al., 2021	1	1	1	1	1	1	1	1	1	1	1	11
Shimizu et al., 2015	1	1	1	1	1	1	1	1	1	1	1	11
Stadheim et al., 2021	1	1	1	1	1	1	1	1	1	1	1	11
Stevenson et al., 2016	1	1	1	1	1	0	0	1	1	1	1	9
Yilmaz et al., 2023	1	1	1	1	1	1	1	1	1	1	1	11
Average												10.65

EC, Eligibility Criteria; RA, Random Allocation, CA, Concealed Allocation; BC, Baseline Comparability, BP, Blind Participants, BT, Blind Therapists, BA, Blind Assessors, AFU, Adequate Follow-Up, ITT, Intention-to-Treat Analysis; BGC, Between-Group Comparisons; PMV, Point Measures and Variability.

The consistently high methodological quality across studies strengthens the credibility of the systematic review's findings. The near-universal implementation of randomization and blinding procedures indicates strong internal validity, while the comprehensive reporting of outcomes enhances the transparency and reproducibility of the research (14, 17, 65, 66). The high rates of participant, therapist, and assessor blinding are particularly noteworthy, as these elements are crucial for minimizing various forms of bias in supplement intervention studies.

**Figure 2:**
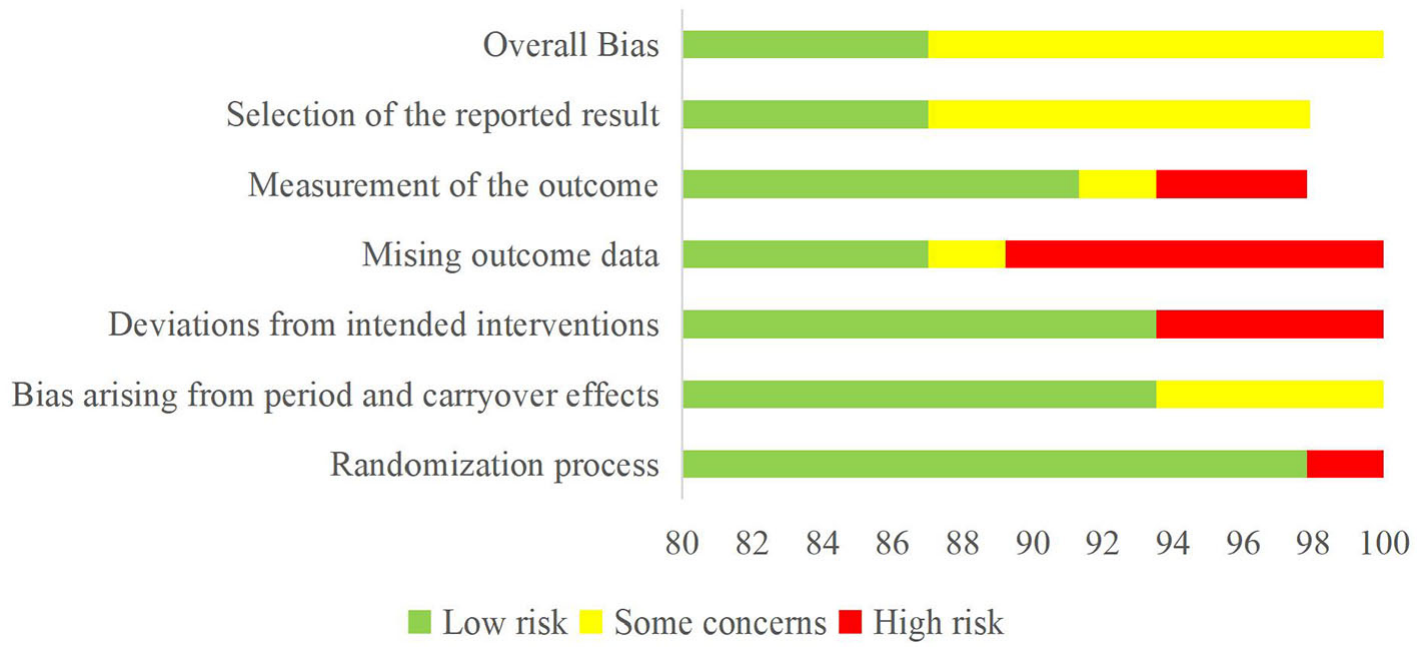
Summary of teh risk of bias.

The risk of bias analysis reveals robust control across multiple domains. Selection bias was effectively minimized through proper randomization and allocation concealment in 98% of studies (see Table 2, Figure 2). Performance and detection bias was well-controlled through comprehensive blinding protocols while reporting bias was addressed through complete outcome documentation. The minor attrition bias risk in 9% of studies, stemming from follow-up and intention-to-treat analysis limitations, represents the primary area for potential methodological improvement in future research. Several factors likely contributed to the high methodological quality observed. First, the studies' recent publication dates (2014–2024) reflect contemporary standards in research methodology. Second, the focus on elite athletes and supplement interventions may have motivated particularly rigorous experimental designs (67). Third, the use of standardized performance measures and controlled testing conditions aligns well with the precise nature of athletic performance assessment.

**Table 2:**
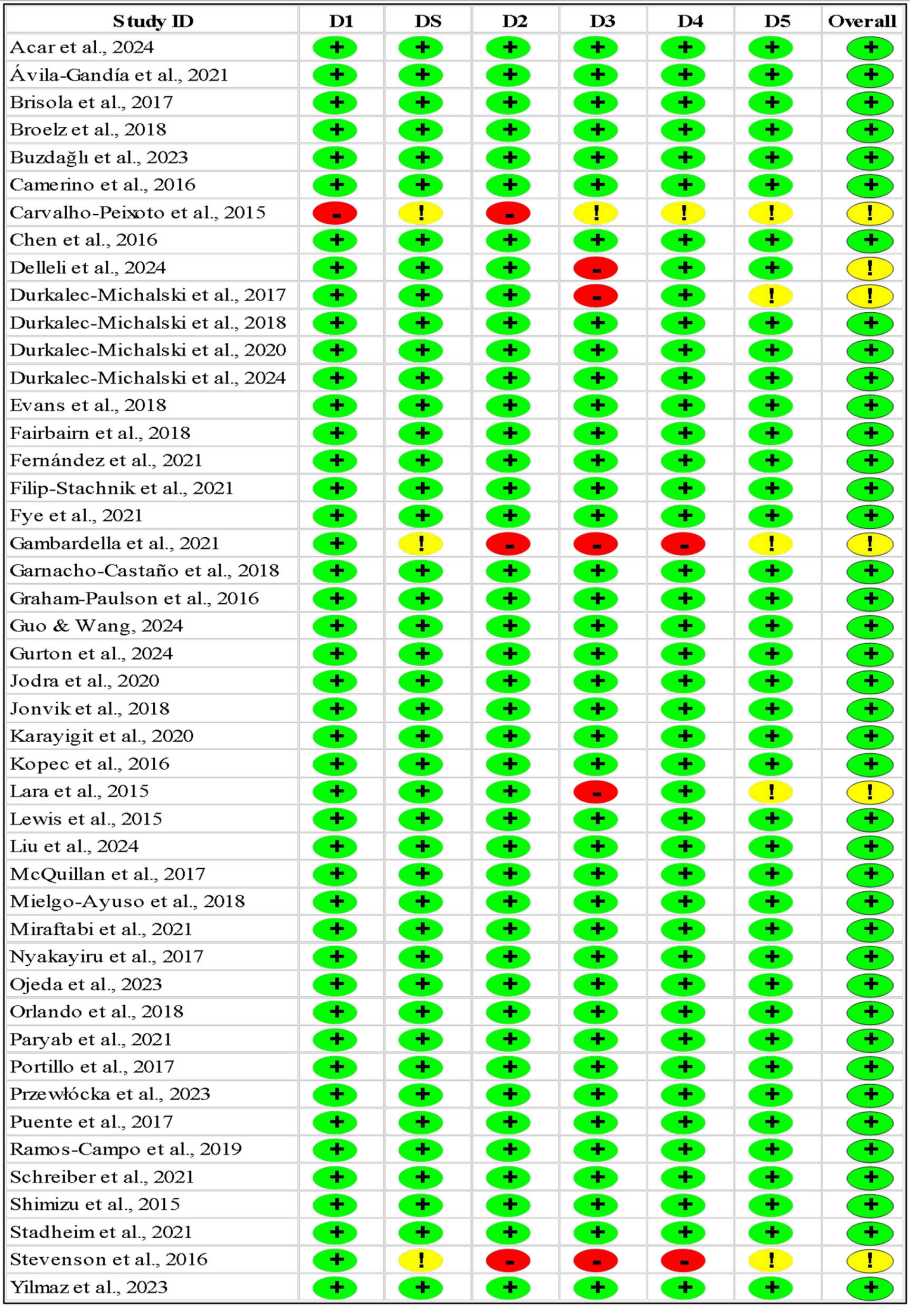
Riskk of bias for every study.

D1, Randomization process; DS, Bias arising from period and carryover effects; D2, Deviations from the intended interventions; D3, Missing outcome data; D4, Measurement of the outcome; D5, Selection of the reported result.

The few studies with lower scores highlight specific methodological challenges in sports supplement research. The difficulties in maintaining perfect follow-up and completing intention-to-treat analyses may reflect the practical challenges of conducting research with elite athletes, including competition schedules and injury-related withdrawals. However, these limitations affected only a small portion of the included studies and did not substantially impact the overall quality of evidence.

### 4.2 Study characteristics

The systematic review encompassed 46 studies (19–64) investigating the efficacy of dietary supplements on the performance of elite athletes, encompassing a total of 928 participants across all studies. The research methodology predominantly featured high-quality experimental designs, with most studies employing double-blind, placebo-controlled trials, randomized and crossover designs. The average study included approximately 20 participants (mean = 20.17), ranging from 8 to 57 participants (see Table 3), reflecting the challenges of recruiting elite athletes for research purposes (68). The studies revealed a significant gender imbalance in supplement research. Approximately 60% of the studies focused exclusively on male athletes (see Table 3), while only 10% studied female athletes exclusively (19, 27, 42, 49, 55). The remaining 30% included both genders (29, 30, 35, 41, 48, 57, 62, 63), highlighting a notable gap in our understanding of supplement effects on female athletes at the elite level; therefore, the results are more biased toward the male gender. The participant age range typically fell between 18 and 40 years, with a mean age across studies of ~24–25 years. The study targeted elite athletes whose average age ranges between 19 and 40 years which are the primary years of athletes; those over 40 years are considered veteran or master athletes (69, 70). This age distribution reflects the prime competitive years for most athletes. Participants generally possessed substantial experience in their respective sports, with most having 5–10 years of competitive experience, ranging from 2 to 15 years.

**Table 3:** Study characteristics of the study.

**Study ID**	**Sport category**	**Sample characteristics**	**Study design**	**Athletic level and experience**
Acar et al., 2024	IND/Swimming	*N* = 8 (8F), 21.3 ± 1.4y	DB-Cross	Club (8.5 ± 1.4y exp)
Ávila-Gandía et al., 2021	END/Cycling	*N* = 11 (11M), 25.5 ± 0.8y	DB-RCT	UCI World Team (Top 5)
Brisola et al., 2017	Team/Water Polo	*N* = 22 (22M), 18.0 ± 4.0y	DB-RCT	Elite (Brazilian Championship)
Broelz et al., 2018	END/Cycling	*N* = 34 (34M), 30.0 ± 5.7y	DB-RCT	Competition (3+ comp/year)
Buzdagli et al., 2023	IND/Speed Skating	*N* = 30 (30M), 23.9 ± 2.8y	DB-Cross	Olympic Prep Team (11.4 ± 3.4y exp)
Camerino et al., 2016	END/Cycling	*N* = 16 (16M), 33.2 ± 2.6y	DB-RCT	National (3y+ exp)
Carvalho-Peixoto et al., 2015	END/Running	*N* = 14 (14M), 26 ± 6y	SB-RCT	Elite (Aeronautical)
Chen et al., 2016	COM/Taekwondo	*N* = 12 (12M), 20.0 ± 0.8y	DB-Cross	National/International (6y+ exp)
Delleli et al., 2024	COM/Taekwondo	*N* = 16 (16F), 18.0 ± 1.0y	DB-Cross	Elite/Black Belt (7y+ exp)
Durkalec-Michalski et al., 2017	COM/Mixed	*N* = 42 (42M), 22.8 ± 6.1y	DB-Cross	Elite (7.3 ± 3.7y exp)
Durkalec-Michalski et al., 2018	COM/Wrestling	*N* = 49 (31M,18F), 19.0 ± 4.0y	DB-RCT	National Team (4y+ exp)
Durkalec–Michalski et al., 2020	COM/Wrestling	*N* = 51 (33M,18F), M:19.7 ± 3.8y, F:18.7 ± 2.4y	DB-RCT	National Team (4y+ exp)
Durkalec-Michalski et al., 2024	END/Swimming/Triathlon	*N* = 28 (28M), 31.1 ± 10.2y	DB-Cross	National (5y+ exp)
Evans et al., 2018	Team/Mixed	*N* = 18 (18M), 21.2 ± 1.1y	DB-Cross	University Level
Fairbairn et al., 2018	Team/Rugby	*N* = 57 (57M), 21.2 ± 2.8y	DB-Cross	Professional (5.5 ± 3.6y exp)
Fernández et al., 2021	COM/Jiu-Jitsu	*N* = 16 (8M,8F), M:21.5 ± 4.8y, F:20.6 ± 3.2y	DB-Cross	National Team (M:11.9 ± 3.9y, F:15.4 ± 2.9y)
Filip-Stachnik et al., 2021	COM/Judo	*N* = 9 (9M), 23.7 ± 4.4y	DB-Cross	International (15.6 ± 4.0y exp)
Fye et al., 2021	END/Running	*N* = 11 (6M,5F), 20.0 ± 2.0y	DB-Cross	NCAA Division I
Gambardella et al., 2021	Team/Water Polo	*N* = 17 (17M), 30.9 ± 1.3y	SB-RCT	Professional (Italian League)
Garnacho-Castaño et al., 2018	END/Cycling	*N* = 12 (12M), 39.3 ± 7.5y	DB-Cross	National/International
Graham-Paulson et al., 2016	Team/Rugby	*N* = 12 (12M), 30.0 ± 7.7y	DB-Cross	Club (6.7 ± 6.0y exp)
Guo & Wang, 2024	Team/Volleyball	*N* = 20 (20M), 24.2 ± 2.6y	DB-RCT	National (8.4 ± 1.8y exp)
Gurton et al., 2024	Team/Soccer	*N* = 10 (10M), 24.0 ± 3.0y	DB-Cross	Collegiate
Jodra et al., 2020	COM/Boxing	*N* = 8 (8M), 22.0 ± 1.8y	DB-Cross	International (2y+ exp)
Jonvik et al., 2018	END/Track Cycling	*N* = 10 (5M,5F), M:21 ± 2y,F:22 ± 3y	DB-Cross	Olympic Track Cyclists
Karayigit et al., 2020	Team/Mixed	*N* = 17 (17F), 23 ± 2y	DB-Cross	Professional League (4 ± 1y exp)
Kopec et al., 2016	Team/Mixed	*N* = 11 (11M), 20 ± 2y	DB-Cross	Club Level
Lara et al., 2015	IND/Swimming	*N* = 14 (14M), 20.2 ± 2.6y	DB-RCT	National (5y+ exp)
Lewis et al., 2015	END/Mixed	*N* = 30 (30M), 35 ± 4.6y	DB-RCT	Olympic Sports (2y+ exp)
Liu et al., 2024	Team/Basketball	*N* = 15 (15M), 20.9 ± 1.0y	DB-Cross	Professional (National Top-8)
McQuillan et al., 2017	END/Cycling	*N* = 9 (9M), 27 ± 9y	DB-Cross	Highly-trained
Mielgo-Ayuso et al., 2018	Team/Volleyball	*N* = 22 (22F), 27.0 ± 5.6y	DB-Cross	Elite
Miraftabi et al., 2021	COM/Taekwondo	*N* = 8 (8M), 20 ± 4y	DB-Cross	National League (>5y exp)
Nyakayiru et al., 2017	END/Cycling	*N* = 17 (17M), 25 ± 4y	DB-Cross	Highly-trained (9.6 ± 5.1y exp)
Ojeda et al., 2023	END/Running	*N* = 12 (12M), 21.8 ± 2.4y	DB-Cross	Competitive (2y+ exp)
Orlando et al., 2018	Team/Rugby	*N* = 21 (21M), 26 ± 5y	DB-Cross	Team Athletes
Paryab et al., 2021	Team/Mixed	*N* = 10 (10M), 20 ± 2y	DB-Cross	Collegiate Championship
Portillo et al., 2017	Team/Rugby Sevens	*N* = 16 (16F), 23 ± 2y	DB-RCT	National Team (4y+ exp)
Przewłócka et al., 2023	COM/MMA	*N* = 23 (23M), 25.5 ± 5.3y	DB-RCT	National (10 ± 4y exp)
Puente et al., 2017	Team/Basketball	*N* = 20 (10M, 10F), M:27.1 ± 4.0y, F:27.9 ± 6.1y	DB-RCT	Pro(F)/Semi-pro(M) (10y+ exp)
Ramos-Campo et al., 2019	END/Running	*N* = 15 (15M), 23.7 ± 8.2y	DB-Cross	International (6y+ exp)
Schreiber et al., 2021	END/Cycling	*N* = 27 (27M), 28.3 ± 5.6y	DB-Cross	Elite/Category 1
Shimizu et al., 2015	COM/Kendo	*N* = 18 (18M), 20.1 ± 1.0y	DB-RCT	Elite Collegiate (13.4 ± 1.5y exp)
Stadheim et al., 2021	END/Mixed	*N* = 23 (23M), 24.0 ± 1.0y	DB-Cross	Elite Endurance
Stevenson et al., 2016	END/Mixed	*N* = 45 (30M, 15F), M:27.1 ± 3.9y, F:27.1 ± 3.2y	SB-RCT	Elite Endurance
Yilmaz et al., 2023	IND/Curling	*N* = 22 (mixed), 20.2 ± 1.6y	DB-Cross	National Team (6.2 ± 0.5y exp)

DB-RCT, Double-blind Randomized Controlled Trial; DB-Cross, Double-blind Crossover; SB-RCT, Single-blind Randomized Controlled Trial; Sports Categories: END (Endurance), COM (Combat), Team, IND (Individual); exp: Experience; N = Sample size; M = Male; F = Female; y = years.

The studies predominantly focused on high-performance athletes where 70% involved elite or professional level athletes; 40% included national team members; 30% featured international competitors; 20% studied national collegiate/university level athletes and 10% examined club-level athletes. In regard to the training regimens; the training volumes were consistently high across studies, reflecting the elite nature of the participants where most athletes trained 3–6 days per week while weekly training hours typically ranged from 6 to 17. Many participants engaged in multiple daily training sessions. The training programs were highly structured and professionally supervised.

The research covered a broad spectrum of sports, which can be categorized into four main groups: endurance, combat, team, and individual sports. The endurance sports featured prominently in the research which included cycling, running, swimming, and triathlon (20, 22, 24, 25, 31, 35, 45, 47, 51, 52, 58, 61, 64); these focused on performance metrics like time to exhaustion and power output. Several studies included compart sports which included wrestling, judo, MMA, taekwondo, and boxing (26–29, 34, 40, 48, 50, 56, 60) which focused more on power and endurance parameters. A number of studies also included team sports with diverse representation including rugby, basketball, volleyball, soccer, and water polo (21, 32, 33, 36–39, 42, 43, 46, 49, 53–55, 57) where the studies individual and team performance metrics. The last category was individual sports where several studies assessed specialized sports like track and field, kendo, curling, and speed skating (19, 23, 41, 44, 59, 62, 63), examining sport-specific performance indicators. Notably, the majority of studies (about 17.4%, n = 8) studies focused on cycling sport (20, 22, 24, 41, 47, 51, 59, 64); four involved rugby (33, 37, 53, 55), only three studies involving swimming (19, 31, 44), running/athletics (25, 35, 52), and taekwondo (26, 27, 50); two studies focusing on wrestling (29, 30), water polo (21, 36), basketball (46, 57) and volleyball (38, 49) while only one studies assessed judo, Mixed Martial Arts (MMA), boxing, jiu-jitsu, kendo, soccer, curling, and speed skating (see Table 3). There tends to be a more focus on assessing dietary supplementation use among endurance athletes more so cycling compared to other sports.

### 4.3 Impact of dietary supplements on performance

#### 4.3.1 Performance enhancers

**Figure 3:**
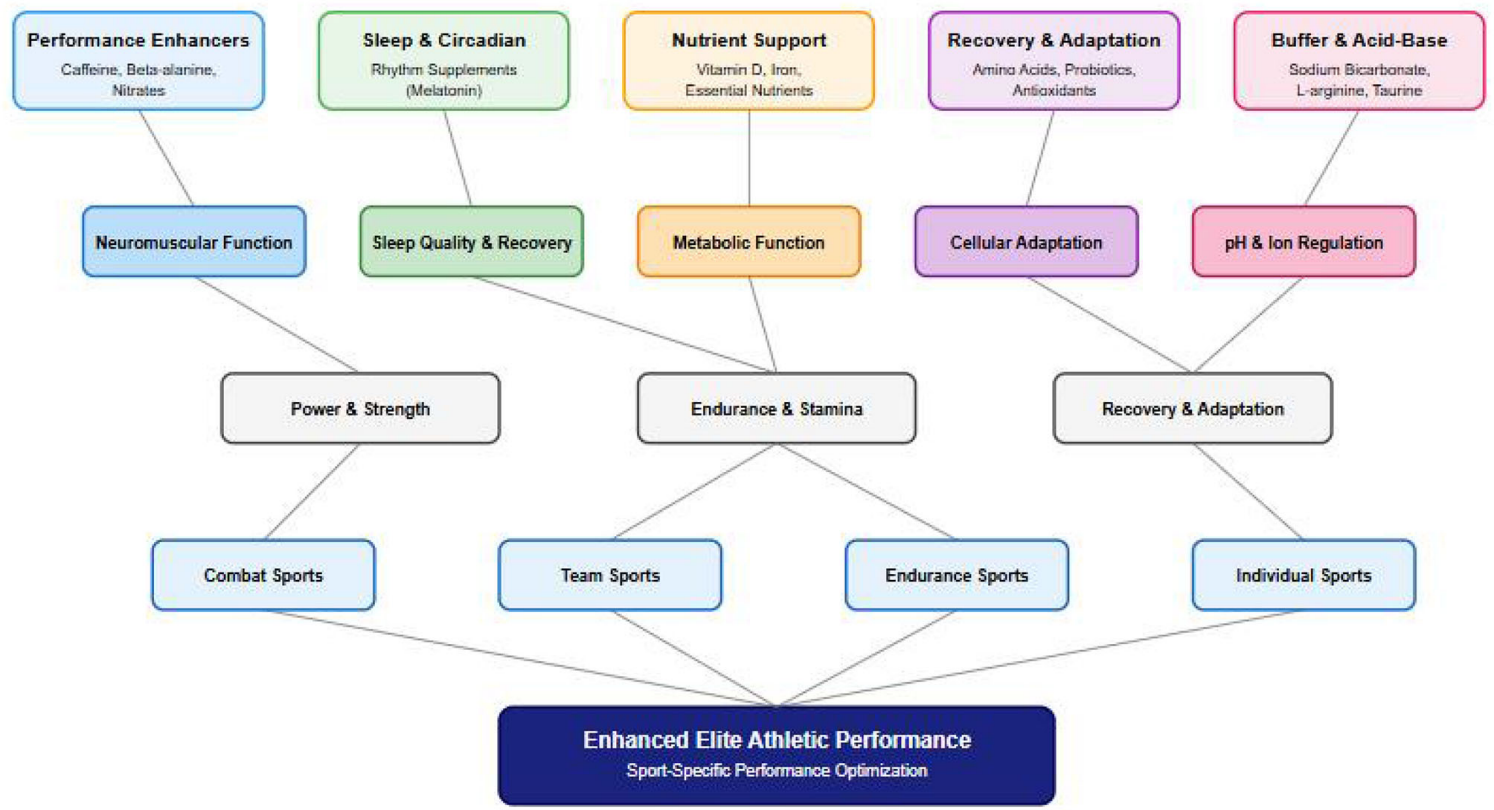
Physiological pathways of dietary supplements in athletic performance.

Out of the 46 articles included in the analysis, 21 (45.7%) articles assessed the impact of performance-enhancing supplements on athlete performance (see Figure 3: Physiological pathways of dietary supplements in athletic performance).

##### 4.3.1.1 Caffeine-based interventions

The efficacy of caffeine supplementation showed notable variation across different sports and performance metrics. In combat sports, Delleli et al. (27) demonstrated significant improvements in technical performance metrics among female taekwondo athletes using a 3 mg/kg dose, highlighting caffeine's potential to enhance both physical and technical aspects of performance. However, Filip-Stachnik et al. (34) found no significant performance improvements in elite judo athletes with doses up to 5.4 mg/kg, suggesting that the ergogenic effects may be sport-specific or influenced by athletes' habitual caffeine consumption. Consequently, in team sports, caffeine supplementation showed more consistent benefits. Puente et al. (57) reported significant improvements in basketball performance metrics, including jump height and game-related statistics, though shooting accuracy remained unchanged. This selective enhancement of certain performance aspects suggests caffeine's effects may be more pronounced in power-based activities rather than precision-based tasks. Similarly, Portillo et al. (55) found increased physical engagement through higher body impact frequency in Rugby Sevens players, though technical skill performance remained unaffected. Recent systematic reviews and meta-analyses further support these findings, indicating that acute caffeine ingestion (3–6 mg·kg^−1^) can enhance sport-specific actions as well as maximal strength and muscular endurance across multiple sports (71, 72).

The timing and context of caffeine administration proved crucial as vindicated by a (58) study which reported no performance improvements in 800 m running when caffeine was administered in the evening, with significant sleep quality impairment noted. This finding underscores the importance of considering the broader impacts of supplementation beyond immediate performance effects. Swimming performance showed particularly promising results, with (19) demonstrating significant improvements in both 25 m and 50 m freestyle times among female swimmers. Similarly, Lara et al. (44) reported enhanced swimming performance metrics including increased jump height and reduced swimming times with caffeinated energy drink consumption.

##### 4.3.1.2 Beta-alanine supplementation

Beta-alanine supplementation showed varying degrees of efficacy across different sports and protocols. One study reported modest improvements in water polo-specific performance parameters after 4 weeks of supplementation, though the between-group differences were not statistically significant (21). This suggests that while beta-alanine may offer some benefits, its effects in team sports settings may be less pronounced than in individual sports. In contrast, (38) demonstrated significant improvements in volleyball players' vertical and horizontal jumps compared to placebo, with enhanced cardiorespiratory fitness and anaerobic power. The divergent findings between these studies might be attributed to differences in loading protocols and performance measures, highlighting the importance of sport-specific supplementation strategies. Another study provided compelling evidence for beta-alanine's efficacy in cycling, showing a 3.43% improvement in time trial performance while the placebo group declined by 6.20% (20). This finding suggests beta-alanine may be particularly effective in preserving performance during intensive training periods. Complementing these sport-specific results, recent evidence indicates that β-alanine—alone or combined with sodium bicarbonate—can improve performance related to the maximal lactate steady state (e.g., endurance-velocity outcomes) (73).

##### 4.3.1.3 Sodium bicarbonate interventions

The implementation of sodium bicarbonate supplementation revealed complex relationships between dosing protocols and performance outcomes. Durkalec-Michalski et al. (29, 30) introduced a novel progressive-dose loading protocol that successfully eliminated gastrointestinal side effects, a common limitation in previous research. However, the performance benefits were limited, with significant improvements observed only in time-to-peak power during the second Wingate test. On the other hand, (39) demonstrated that oral sodium bicarbonate supplementation (0.3 g/kg) improved repeated sprint performance in soccer players, particularly during half-time and post-exercise periods. This finding suggests that the timing of supplementation may be as crucial as the dosing protocol. Synthesizing across studies, umbrella-level evidence indicates that pre-exercise sodium bicarbonate produces small but consistent improvements in high-intensity and repeated-sprint performance, with effect magnitude moderated by dose and gastrointestinal tolerance (74).

##### 4.3.1.4 Nitrate supplementation

The efficacy of nitrate supplementation in elite athletes presented a complex picture just like other performance-enhancing dietary supplements. Nyakayiru et al. (51) found that despite significantly increased plasma nitrate and nitrite concentrations, both acute and 6-day supplementation protocols failed to improve submaximal exercise efficiency or time-trial performance in highly-trained cyclists. This finding aligns with (47) who reported that short-term nitrate supplementation actually impaired 1 km time trial performance. Garnacho-Castaño et al. (64) similarly found no significant improvements in cardio ventilatory responses or mechanical exercise economy in well-trained triathletes. These consistent findings across multiple studies (75–77) suggest that highly-trained athletes may have reduced sensitivity to nitrate supplementation, possibly due to training-induced physiological adaptations. Consistent with this, recent umbrella reviews and meta-analyses indicate that the ergogenic effects of dietary nitrate are task-dependent and moderated by training status, with notably less consistent benefits in highly trained athletes (78).

#### 4.3.2 Sleep nutrient support and sleep and circadian rhythm supplements

Three studies were categorized in this section which contained melatonin classified a sleep and circadian rhythm supplement (54) while Vitamin D and *Ferrous sulfate* (iron) supplements were categorized as nutrient support supplements (33, 49) (see Table 4). The efficacy of nutrient support and sleep-related supplements in elite athletes presented results with varying degrees of effectiveness across different interventions. Vitamin D supplementation (50,000 IU fortnightly, equivalent to 3,570 IU/day) demonstrated limited ergogenic benefits in professional rugby players despite significantly increasing serum 25(OH)D concentrations (33). While the supplementation protocol successfully elevated vitamin D status by 32 nmol/L compared to placebo (*p* < 0.001), only weighted reverse-grip chin-up performance showed significant improvement (+5.5 kg, 95% CI: 2.0 to 8.9, *p* = 0.002). Consistent with this selective pattern, updated meta-analyses report that vitamin D3 supplementation can improve lower-limb muscle strength in athletes, with larger effects in those deficient at baseline (79). This selective performance enhancement raises questions about the mechanism of action and suggests that vitamin D's ergogenic effects may be more specific than previously thought, particularly in athletes with adequate baseline status.

**Table 4:** Results on the impact of nutrient support and sleep & circadian rhythm supplements on athlete performance.

**Category**	**Study**	**Sport**	**Supplement**	**Training metrics**	**Physiological markers**
Sleep and circadian rhythm	Paryab et al. (2020)	Student-athletes	Melatonin	Improved sleep-deprived performance	Not reported
Nutrient support supplements	Fairbairn et al. (2018)	Rugby	Vitamin D	Improved weighted chin-up	Increased serum 25(OH)D
	Mielgo-Ayuso et al. (2018)	Volleyball	Ferrous sulfate	Power improvements maintained	Higher serum iron

All results reported are statistically significant unless otherwise noted.

Iron supplementation in elite female volleyball players (325 mg/day *Ferrous sulfate* for 11 weeks) showed initial benefits that weren't sustained after supplementation cessation (49). The transient nature of these improvements suggests continuous supplementation throughout the competitive season may be necessary for maintaining performance benefits. This finding has important implications for long-term supplementation strategies in female athletes, particularly in iron-demanding sports. The investigation of sleep and circadian rhythm modulation through melatonin supplementation (6 mg) showed promising results in sleep-deprived athletes (54). The supplement significantly improved psychomotor and physical performance measures following both 4-h and 24-h sleep deprivation, though notably showed no benefits under normal sleep conditions. This context-specific efficacy suggests melatonin's potential as a targeted intervention for competition scenarios involving disrupted sleep patterns rather than as a general performance enhancer.

When examining combined nutrient approaches, the integration of probiotics with vitamin D (2 × 10∧9 CFUs probiotics + 3,000–4,000 IU vitamin D_3_ daily) demonstrated synergistic benefits in MMA athletes (56). This combination improved total work output and mean power during high-intensity exercise while enhancing lactate utilization, suggesting that multi-nutrient approaches may offer advantages over single-nutrient supplementation strategies. Moreover, multiple systematic reviews indicate that probiotics may indirectly support training adaptations and competitive performance by improving gastrointestinal comfort, immune function, and fatigue-related markers (80–82). Critical analysis reveals several important considerations in interpreting these results. First, the effectiveness of nutrient supplementation appears highly dependent on baseline status, as demonstrated by the limited benefits of vitamin D supplementation in athletes with adequate levels. Second, the temporal aspects of supplementation emerge as crucial factors, with benefits often requiring sustained intervention and potentially dissipating upon cessation. Third, the specificity of performance improvements suggests that nutrient supplementation may need to be more precisely targeted based on both sports demands and individual athlete characteristics.

These findings have significant implications for practical application. The data suggests that blanket supplementation approaches may be less effective than targeted interventions based on documented deficiencies or specific performance demands. Additionally, the timing and duration of supplementation emerge as critical factors that need careful consideration in supplement protocols. The variable responses observed across different studies also highlight the importance of individual monitoring and adjustment of supplementation strategies.

#### 4.3.3 Recovery and adaptation supplements

The research on recovery and adaptation supplements encompassed several key categories, each showing varying degrees of efficacy in elite athletes (see Table 5).

**Table 5:** Results on the impact of recovery & adaptation dietary supplements on athlete performance.

**Study**	**Sport**	**Supplement**	**Training metrics**	**Physiological markers**
Broelz et al. (2018)	Cycling	BCAA mixture	Improved Individual Anaerobic Threshold	Increased blood lactate
Camerino et al. (2016)	Cycling	Keto analogs and amino acids	No significant endurance differences	Changes in ammonia response
Carvalho-Peixoto et al. (2015)	Running	Açai beverage	Improved time to exhaustion	Better blood markers
Chen et al. (2016)	Taekwondo	BCAA + Arginine + Citrulline	Improved reaction times	Higher plasma BCAA
Durkalec-Michalski et al. (2024)	Swimming/triathlon	Colostrum	No significant performance differences	Reduced post-exercise markers
Evan Lewis et al. (2015)	Olympic sports	Seal oil N-3 PUFA	Reduced power drop	Increased plasma EPA
Orlando et al. (2018)	Rugby	Ubiquinol	Not reported	Reduced oxidative stress
Przewłócka et al. (2023)	MMA	Probiotics + Vitamin D	Total work improved	Lower lactate concentrations
Schreiber et al. (2021)	Cycling	Probiotics	No VO2max changes	Reduced GI symptoms
Shimizu et al. (2015)	Kendo	Coenzyme Q10	Not reported	Higher serum CoQ10
Stevenson et al. (2016)	Endurance sports	Echinacea	Mixed VO2max responses	No significant blood changes

All results reported are statistically significant unless otherwise noted.

##### 4.3.3.1 Amino acid-based supplements

Branched-Chain Amino Acids (BCAAs) and related compounds demonstrated mixed effects on performance and recovery. A notable study found that combined supplementation of BCAAs, arginine, and citrulline effectively prevented exercise-induced central fatigue in elite taekwondo athletes, evidenced by maintained premotor reaction times and improved secondary task performance compared to placebo (26). The mechanism appeared to be related to lower tryptophan/BCAA ratios and increased nitric oxide production without additional ammonia accumulation. Similarly, a BCAA mixture improved Individual Anaerobic Threshold performance in cyclists, though this was accompanied by increased blood lactate levels, suggesting enhanced metabolic capacity rather than improved efficiency (22). However, not all amino acid interventions showed clear benefits. The administration of keto analogs and amino acids (KAAA) successfully reduced exercise-induced blood ammonia increases but failed to demonstrate significant effects on physical performance or cognitive-motor tasks in cyclists (24). Consistent with the broader amino acid literature, systematic reviews and randomized controlled trials also indicate that L-arginine can improve metabolic responses and select performance outcomes in high-intensity, sport-specific tests, with effects depending on dose, timing, and sport context (83, 84). This suggests that while amino acid supplementation may improve certain physiological markers, these improvements do not necessarily translate to enhanced performance outcomes.

##### 4.3.3.2 Probiotic and gut health interventions

Recent research has shown promising results for probiotic supplementation, particularly when combined with other nutrients. A 4-week combined supplementation of probiotics and vitamin D_3_ enhanced lactate utilization and improved anaerobic performance in MMA athletes, with the intervention group showing better total work output and mean power during high-intensity exercise (56). The supplementation also improved lactate clearance without any reported adverse effects. A longer-term study (59) over 90 days found that while probiotic supplementation didn't enhance direct performance measures in elite cyclists, it significantly reduced gastrointestinal symptoms and perceived exertion during exercise. This suggests that probiotics may contribute to performance indirectly by improving exercise tolerance and recovery through reduced GI distress (80).

##### 4.3.3.3 Antioxidant and cellular health supplements

Several studies investigated supplements targeting cellular health and oxidative stress. Coenzyme Q10 (CoQ10) supplementation showed effectiveness in modulating inflammatory responses during intensive training. In elite kendo athletes, CoQ10 successfully suppressed the increase of inflammatory markers (TLR-4+/CD14+ cells) during intensive training compared to placebo (60). Similarly, ubiquinol supplementation (200 mg/day) enhanced plasma and cellular antioxidant defenses in rugby players, though it didn't improve physical performance or prevent exercise-induced muscle damage markers (53). Evidence-based reviews further indicate that CoQ10—including ubiquinol—can improve oxidative stress and certain performance-related outcomes, but overall effects appear to be moderated by heterogeneity in populations and dosing (85, 86). The research on Echinacea-based supplements (EBS) proved less promising. Neither regular (8,000 mg/day) nor double (16,000 mg/day) doses improved VO2max or blood parameters in endurance-trained athletes compared to placebo, suggesting limited utility for performance enhancement in trained athletes (62). The results show a lack of uniformity in the efficacy of antioxidant and cellular health supplements.

##### 4.3.3.4 Natural products and omega-3 supplements

Research on natural product supplements showed varying degrees of success similar to other recovery and adaptation supplements. An açai-based beverage intervention significantly increased time to exhaustion during high-intensity exercise and attenuated exercise-induced metabolic stress (25). The beverage improved effort tolerance and reduced physiological markers of fatigue, though the short intervention period and small sample size warrant careful interpretation of these results. In swimming and triathlon athletes, Colostrum Bovinum supplementation (25 g/day for 12 weeks) showed no significant ergogenic effect on swimming performance compared to placebo, though both supplements improved exercise adaptation markers through reduced blood lactate (31). Evidence from randomized controlled trials indicates that omega-3 supplementation helps attenuate post-exercise inflammation and muscle damage and may facilitate performance recovery (87).

N-3 PUFA supplementation demonstrated promising results for neuromuscular function. A 21-day supplementation protocol improved peripheral neuromuscular function and reduced fatigue during sprint cycling, though effects on central neuromuscular function remained unclear (45). The improvements were associated with significant increases in plasma EPA levels, suggesting successful supplement absorption and utilization.

#### 4.3.4 Buffer and acid-base supplements

The research on buffer and acid-base supplements revealed multifaceted interactions between supplementation protocols and performance outcomes, with notable variations in efficacy across different sporting contexts and populations (see Table 6).

**Table 6:** Results on the impact of buffer & acid-base supplements on athlete performance.

**Study**	**Sport**	**Supplement**	**Training metrics**	**Physiological markers**
Durkalec-Michalski et al. (2017)	Combat sports	β-hydroxy-β-methylbutyrate (HMB)	Increased anaerobic power	Improved body composition
Durkalec-Michalski et al. (2018)	Wrestling	Sodium bicarbonate	Improved peak power timing	No metabolic differences
Durkalec-Michalski et al. (2020)	Wrestling	Sodium bicarbonate	No significant power differences	No metabolic differences
Gambardella et al. (2021)	Water Polo	L-Arginine	No significant speed differences	Lower lactate-to-speed ratio
Kopec et al. (2015)	Team sports	Sodium Phosphate + Caffeine	Improved sprint times	No blood marker changes
Buzdagli et al. (2023)	Various	Taurine	Improved power metrics	Higher lactate, lower RPE

All results reported are statistically significant unless otherwise noted.

##### 4.3.4.1 Sodium bicarbonate

Research on sodium bicarbonate supplementation demonstrated effects that varied based on dosing protocols and athlete characteristics. A novel progressive-dose loading protocol, starting at 25 mg/kg/day and increasing to 100 mg/kg/day over 10 days as demonstrated by Durkalec-Michalski et al. (29), successfully eliminated the common gastrointestinal side effects associated with acute sodium bicarbonate loading. However, this protocol showed limited performance benefits, with the only significant improvement being a 32% reduction in time-to-peak power during the second Wingate test (29). This finding suggests that while the protocol improved tolerability, the conservative dosing approach may have compromised potential performance benefits. Gender-specific responses to sodium bicarbonate supplementation emerged as a significant consideration. In wrestling performance, male athletes demonstrated meaningful improvements in sport-specific tasks such as dummy throws, while female athletes showed no significant performance enhancement (30). These gender differences highlight the importance of considering physiological variations in supplement response, potentially related to differences in muscle fiber composition and glycolytic capacity between males and females. Recent research in soccer demonstrated that oral sodium bicarbonate supplementation (0.3 g/kg) effectively improved repeated sprint performance, particularly during half-time and post-exercise periods (39). The enhanced performance was attributed to improved blood buffering capacity and better regulation of strong ions. Notably, when comparing administration methods, oral supplementation proved significantly more effective than topical application, despite equivalent sodium bicarbonate content.

##### 4.3.4.2 L-Arginine and taurine supplementation

L-Arginine supplementation (5 g/day) demonstrated interesting metabolic effects in water polo players, though not directly translate to performance enhancement (36). While maximal performance measures remained unchanged, the supplementation led to significantly lower lactate-to-speed ratios, indicating improved oxidative metabolism during exercise. This improvement appeared to be mediated through enhanced mitochondrial function and biogenesis. The findings suggest that L-Arginine's benefits may be more subtle, enhancing metabolic efficiency rather than directly improving performance metrics. Acute taurine supplementation showed promising results in speed skaters, with significant enhancements in anaerobic power outputs without corresponding increases in neuromuscular fatigue (23). The intervention demonstrated improved peak power, mean power, and minimum power during Wingate testing compared to placebo. Interestingly, while blood lactate levels were elevated with taurine supplementation, perceived exertion was lower, suggesting enhanced tolerance to high-intensity exercise.

##### 4.3.4.3 BCAA and amino acid combinations

Research examining BCAA mixtures in cycling revealed that supplementation improved Individual Anaerobic Threshold performance, accompanied by increased blood lactate levels. This finding suggests enhanced buffering capacity or improved lactate tolerance, though the exact mechanism remains unclear (22). Similarly, the investigation of keto analogs and amino acids (24) showed reduced exercise-induced blood ammonia increases, though this did not translate to significant improvements in physical performance or cognitive-motor tasks. Recent meta-analyses further indicate that BCAA supplementation can reduce exercise-induced muscle damage and soreness and, in some studies, accelerate functional recovery (88). This suggests that while amino acid supplementation may improve certain physiological markers, these improvements do not necessarily translate to enhanced performance outcomes.

##### 4.3.4.4 Sodium phosphate combined with caffeine

The examination of sodium phosphate supplementation in team sports athletes, both alone and in combination with caffeine, revealed modest improvements in repeated sprint ability compared to placebo, with moderate to large effect sizes (43). However, the combined protocol (SP+C) showed no additional benefits over sodium phosphate alone, suggesting limited synergistic effects between these supplements.

The findings across these studies underscore the complexity of buffer and acid-base supplementation in elite sports. While some protocols show promise, the results emphasize the importance of considering individual response variations, gender differences, and sport-specific demands in supplementation strategies. The research also highlights the need for careful protocol design to balance efficacy with tolerability, particularly for supplements known to cause gastrointestinal distress.

#### 4.3.5 Dual-purpose supplements: evidence for concurrent performance enhancement and recovery

Synthesizing the RCTs included in this review together with recent systematic reviews and guideline statements, several “dual-purpose” supplements appear to both enhance acute/short-term performance and accelerate post-event recovery. Representative agents include caffeine, β-alanine, whey/casein proteins, and—under certain conditions—BCAAs, antioxidants/polyphenols/coenzyme Q10, and probiotics (± vitamin D). Specifically, caffeine improves central arousal and neuromuscular drive via adenosine-receptor antagonism, consistently enhancing endurance, sprinting, and sport-specific skill execution while lowering ratings of perceived exertion and pain/soreness (3–6 mg/kg ingested 30–60 min pre-exercise; co-ingestion with carbohydrate may also facilitate post-exercise muscle glycogen resynthesis) (89–91). β-Alanine raises intramuscular carnosine to augment H^+^ buffering, thereby increasing mean power and tolerance during 1–10 min high-intensity/lactate-dominant efforts and, through buffering and possible antioxidant effects, attenuating post-exercise discomfort (4–6 g/day, ≥2–4 weeks loading) (92). Protein timing likewise exhibits dual effects: post-exercise whey (20–30 g) rapidly stimulates muscle protein synthesis and shortens functional recovery time; pre-sleep casein (40–48 g) provides overnight amino-acid delivery to maintain a positive nitrogen balance, improving next-day performance and longer-term strength/lean-mass adaptations (93, 94). These conclusions are corroborated by RCT evidence in elite cycling, volleyball, swimming, basketball, and combat sports included herein, supporting prioritization of these strategies during competition phases and high-intensity training weeks.

By contrast, the mean effect of BCAAs on immediate performance is small; however, sustained supplementation (≥5 g per dose for >7 days) can significantly lower serum creatine kinase and delayed-onset muscle soreness, thereby shortening the recovery window—effects that are particularly valuable when total protein intake is suboptimal or recovery demands are heavy (95). Antioxidants/polyphenols/coenzyme Q10 can mitigate oxidative stress and inflammation, reduce DOMS, and maintain training availability during congested schedules, but dosing and timing should be managed to avoid blunting training adaptations with chronic high-dose use (96–98). Probiotics (10∧9–10∧10 CFU/day for 4–12 weeks) show less consistent effects on “immediate performance,” yet reduce GI symptoms and infection risk, lower perceived exertion, and improve training tolerance; when combined with vitamin D, greater lactate clearance and anaerobic output have been observed in combat-sport/high-intensity interval contexts (80, 82, 99). Given sport specificity, training status, and inter-individual variability, these supplements should be implemented within an individualized, periodized framework: e.g., use caffeine for event/class “peaking,” β-alanine during the pre-competition phase to build buffering capacity, whey/casein for post-exercise and nocturnal anabolic support, and BCAAs with judicious antioxidants/polyphenols and probiotics to bolster tolerance and health during heavy-load weeks. Coupled with carbohydrate management, adequate total protein, and sleep hygiene, such integration maximizes the composite benefits of “dual-purpose” supplementation (80, 82, 89–99).

Figure 3 presents a comprehensive conceptual framework illustrating how dietary supplements influence elite athletic performance through distinct physiological pathways. This model reflects the complex landscape of sports supplementation, where usage among elite athletes typically ranges from 40% to 100%, varying based on sport type, competition level, and athlete characteristics (7, 67). The framework encompasses five primary supplement categories that align with current understanding of performance enhancement strategies in elite sports (1, 3). Performance enhancers, including caffeine, beta-alanine, and nitrates, primarily influence neuromuscular function and acute performance capabilities. These supplements have shown consistent evidence in enhancing power output and technical performance, particularly in competitive environments where marginal gains can significantly impact outcomes (6, 12). Sleep and circadian rhythm supplements, anchored by melatonin, represent an emerging category addressing the critical role of recovery optimization in elite performance, reflecting the growing recognition of sleep quality as a key performance determinant (1, 3, 12).

Nutrient support supplements, including vitamin D and iron, maintain fundamental metabolic functions essential for sustained high-level performance. This category addresses the increased nutritional demands of elite training, where dietary supplementation often becomes necessary to support optimal physiological function (1, 3, 12). Recovery and adaptation supplements, comprising amino acids, probiotics, and antioxidants, enhance cellular adaptation and repair processes. These supplements reflect the evolution in understanding of recovery dynamics in elite sports, where traditional recovery methods are increasingly complemented by targeted supplementation strategies (1, 3, 7, 12). Buffer and Acid-Base Supplements, including sodium bicarbonate, L-arginine, and taurine, play a crucial role in regulating physiological homeostasis during intense exercise. Their effectiveness varies across different sporting contexts, highlighting the importance of sport-specific supplementation protocols (13, 100, 101). The model demonstrates how these supplement categories operate through distinct yet interconnected physiological mechanisms, converging on three key performance outcomes: power and strength, endurance and stamina, and recovery adaptation.

The framework's final level addresses sport-specific applications, recognizing that supplement effectiveness varies significantly between combat sports, team sports, endurance events, and individual disciplines. This sport-specific consideration is crucial, as research indicates that supplementation strategies must be tailored to both the physiological demands of different sports and individual athlete characteristics (7, 12, 102, 103). This integrated model serves as a foundation for analyzing the evidence presented in this systematic review, while acknowledging the complexity of supplement interactions and sport-specific demands. It provides a structured approach for understanding how different supplements might benefit athletes across various sporting contexts, supporting evidence-based decision-making in elite sport nutrition strategies.

### 4.4 Effect of dosing protocols

The efficacy of dietary supplements showed significant dependence on dosing strategies, with timing, quantity, and administration protocols playing crucial roles in performance outcomes (see Tables 6, 7).

**Table 7:** Summary of supplementation protocols across all studies.

**Study**	**Supplement name**	**Dosage protocol**	**Intervention duration**
Acar et al. 2024	Caffeine	Daily Dose: 6 mg/kg body mass caffeine; Single dose 60 min before exercise	Acute supplementation
Ávila-Gandía et al. 2021	β-alanine	20g β-alanine; 4 times daily (5g per dose)	Acute supplementation
Brisola et al. 2016	β-alanine	Daily Dose: First 10 days: 4.8g/day (6x800mg); Final 18 days: 6.4g/day (4x1600mg); Loading: Two-phase loading; Frequency: Multiple daily doses	4 weeks
Broelz et al. 2018	BCAA mixture	Daily Dose: 4.3g BCAA (1.5g Valin, 1.3g Leucine, 1.5g Isoleucine)	Acute supplementation
Buzdagli et al. 2023	Taurine	Daily Dose: 6g single dose; Mixed with 500ml water; Frequency: 60 min before testing	Single acute trial
Camerino et al. 2016	Keto analogs and amino acids	5 tablets containing amino acid mixture; Single dose with ~300 mL water; 1 hour before exercise	Acute supplementation
Carvalho-Peixoto et al. 2015	Açai beverage	300mL containing 27.6mg anthocyanins; Loading: 3 consecutive days; Frequency: Once daily plus pre-exercise dose	3 days + pre-exercise
Chen et al. 2016	BCAA + Arginine + Citrulline	BCAA: 0.17 g/kg; Arginine: 0.05 g/kg; Citrulline: 0.05 g/kg	Acute supplementation
Delleli et al. 2024	Caffeine	3 mg·kg−1 body mass	Acute supplementation
Durkalec-Michalski et al. 2017	HMB	Daily Dose: 3 g (1000 mg per capsule, taken as three capsules daily); Frequency: Upon waking, after training, before sleep	12 weeks
Durkalec-Michalski et al. 2018	Sodium bicarbonate	Progressive loading: Days 1-2: 25 mg/kg/day to Days 8-10: 100 mg/kg/day; Three evenly split doses	10 days
Durkalec-Michalski et al. 2020	Sodium bicarbonate	Progressive loading (25-100 mg/kg/day); Three daily doses	10 days
Durkalec-Michalski et al. 2024	Colostrum	25g/day total (Split into two 12.5g doses)	12 weeks
Evans et al. 2017	Caffeine gum	200 mg caffeine (2 sticks providing 100 mg each); Chewed for 10 min prior	Acute supplementation
Fairbairn et al. 2018	Vitamin D	50,000 IU (1.25mg) equivalent to 3,570 IU/day; Frequency: Once every fortnight	11-12 weeks
Fernández et al. 2021	Caffeine	Daily Dose: 3 mg/kg body mass	Acute supplementation
Filip-Stachnik et al. 2021	Caffeine gum	Two protocols: C+P: 200 mg; C+C: 400 mg; 15 min before exercise	Acute supplementation
Fye et al. 2021	Multi-ingredient pre-workout	10g containing beetroot powder, taurine, beta-alanine, caffeine	Acute supplementation
Gambardella et al. 2021	L-Arginine	5 grams per day	Not specified
Garnacho-Castaño et al. 2018	Beetroot juice	70 ml (~6.5 mmol, 404 mg of NO3-); 3 hours before test	Acute supplementation
Graham-Paulson et al. 2016	Caffeine	4 mg/kg anhydrous caffeine; 70 min before performance	Acute supplementation
Guo et al. 2024	Beta-alanine	4.8g total divided into six equal doses of 0.8g; Every two hours throughout the day	8 weeks
Gurton et al. 2024	Sodium bicarbonate	SB-ORAL: Three separate 0.1 g/kg doses at 15 min intervals; SB-LOTION: Three separate 0.3012 g/kg doses	Acute supplementation
Jodra et al. 2020	Caffeine	6 mg/kg body weight; 60 min before testing	Acute supplementation
Jonvik et al. 2018	Beetroot juice	140 mL beetroot juice daily; 6-day supplementation period	6 days
Karayigit et al. 2021	Caffeine	Doses: 3mg/kg and 6mg/kg body mass; 60 min before testing	Acute supplementation
Kopec et al. 2016	Sodium Phosphate + Caffeine	SP: 50 mg/kg free fat mass daily for 6 days; Caffeine: 6 mg/kg body mass	14 weeks
Lara et al. 2015	Caffeinated energy drink	3 mg/kg of body mass; Ingested 60 min before trials	Single acute trial
Lewis et al. 2015	Seal oil N-3 PUFA	5 mL (375 mg EPA, 230 mg DPA, 510 mg DHA, 1000 IU vitamin D3); 2-2.5 mL servings twice daily	21 days
Liu et al. 2024	Caffeine gum	3 mg/kg of body weight; 10 min chewing followed by 15 min rest	Acute supplementation
McQuillan et al. 2017	Beetroot juice	140 mL containing ~8.0mmol NO3-; 2.5hr before time trials	Acute and chronic (6-7 days)
Mielgo-Ayuso et al. 2018	Ferrous sulfate	325 mg/day ferrous sulfate (105 mg/day elemental iron)	29 weeks
Miraftabi et al. 2021	Beetroot juice	BJ-400: 60mL BJ (400mg NO3-) + 60mL placebo; BJ-800: 120mL BJ (800mg NO3-)	Acute supplementation
Nyakayiru et al. 2017	Sodium nitrate	1097 mg sodium nitrate (~12.9 mmol nitrate/day)	Acute (1-day) and chronic (6-day)
Ojeda et al. 2023	Beta-alanine	Low dose: 30 mg/kg body mass; High dose: 45 mg/kg body mass	Acute supplementation
Orlando et al. 2018	Ubiquinol	200 mg ubiquinol/day; Once daily with meals	1 month
Paryab et al. 2021	Melatonin	6 mg melatonin; 30 min before exercise testing	Acute supplementation
Portillo et al. 2017	Caffeine	3 mg of caffeine per kg of body mass; 60 min before competition	Acute supplementation
Przewłócka et al. 2023	Probiotics + Vitamin D	Probiotics: 4 capsules daily (2 × 109 CFUs total); Vitamin D3: 3–4 drops daily (3,000–4,000 IU)	4 weeks
Puente et al. 2017	Caffeine	3 mg/kg body mass; 60 min before testing	Acute supplementation
Ramos-Campo et al. 2019	Caffeine	6 mg/kg body mass; 60 min before evening testing	Acute supplementation
Schreiber et al. 2021	Probiotics	~15 billion CFU daily; One capsule per day	90 days
Shimizu et al. 2015	Coenzyme Q10	300 mg (3 × 100 mg capsules) per day; Once daily in the morning after breakfast	20 days
Stadheim et al. 2021	Caffeine	4.5 mg·kg−1; 45 min before standardized warm-up	2 weeks
Stevenson et al. 2016	Echinacea	RD group: 8,000 mg/day; DD group: 16,000 mg/day; Four times daily	35 days
Yilmaz et al. 2023	Caffeine + L-theanine	CAF: 6 mg/kg; THE: 6 mg/kg; CAFTHE: 6 mg/kg CAF + 6 mg/kg THE	Acute supplementation

Acute supplementation typically refers to single-dose administration before testing or performance assessment. Where specific timing information was available, it has been included in the dosage protocol.

#### 4.4.1 Acute vs. chronic supplementation

Acute supplementation proved effective for certain ergogenic aids but showed limitations for others. Caffeine demonstrated consistent benefits with acute dosing of 3–6 mg/kg body mass when administered 45–60 min pre-exercise, improving sprint performance, power output, and technical skills (37, 40, 42, 44, 46, 57). However, acute beetroot juice supplementation (70–140 ml containing 6.5–8.0 mmol nitrate) showed limited benefits in elite athletes, suggesting that highly-trained individuals might require different dosing strategies (41, 47, 51, 64). Chronic supplementation protocols showed varying effectiveness depending on the supplement type. Beta-alanine supplementation using a progressive loading protocol (4.8 g/day for the first 10 days, increasing to 6.4 g/day for the final 18 days) demonstrated significant improvements in power output and performance metrics (21, 38, 52). Similarly, long-term iron supplementation (325 mg/day ferrous sulfate) showed benefits in female athletes, though these improvements weren't maintained after supplementation ceased (49) while probiotic supplementation (56, 59) (15 billion CFU daily for 90 days) improved exercise tolerance without enhancing direct performance measures. The evidence suggests that while acute supplementation protocols are effective for immediate performance enhancement with certain supplements (particularly caffeine), chronic loading protocols tend to provide more consistent and sustainable benefits for supplements affecting physiological adaptation pathways, such as beta-alanine and iron, though maintaining these benefits often requires continuous supplementation throughout the competitive season.

#### 4.4.2 Progressive loading protocols and timing considerations

Progressive loading emerged as an effective strategy for minimizing side effects while maintaining ergogenic benefits. Sodium bicarbonate loading protocols starting at 25 mg/kg/day and increasing to 100 mg/kg/day over 10 days successfully eliminated gastrointestinal side effects while improving performance metrics (29, 39). Similarly, beta-alanine loading protocols using sustained-release formulations allowed higher daily doses (20 g) without the typical paraesthesia side effects (20). Progressive loading protocols emerge as a superior strategy for supplements known to cause side effects, effectively balancing ergogenic benefits with tolerance, particularly evident in sodium bicarbonate and beta-alanine supplementation, though this approach requires longer preparation periods and careful monitoring of individual responses.

Supplement timing significantly influenced efficacy. Caffeine supplementation showed optimal effects when administered 60 min pre-exercise for most performance metrics (27, 48, 55). However, evening administration of caffeine (6 mg/kg) before 800 m running performance showed no benefits while significantly impairing sleep quality (58). Nitrate supplementation (50, 51) required longer pre-exercise timing (2.5–3 h) but showed limited benefits in elite athletes regardless of timing. The timing of supplement administration proves to be a critical factor in maximizing ergogenic benefits, with optimal windows varying significantly between supplements and performance contexts, highlighting the need for sport-specific and circadian-aware supplementation strategies rather than one-size-fits-all approaches.

#### 4.4.3 Sport-specific dosing

Different sports require varied dosing strategies for optimal results. Combat sports athletes showed better responses to moderate caffeine doses (3–4 mg/kg) for improving both physical and technical performance (26, 34, 48). Team sports benefited from varied protocols: caffeine (3–6 mg/kg) improved game-related statistics and physical performance (32, 57), while beta-alanine and sodium bicarbonate (21, 29) required more structured loading protocols for optimal effects. The varying effectiveness of dosing protocols across different sports underscores the necessity of tailoring supplementation strategies to specific sporting demands and individual athlete characteristics, with combat sports and team sports in particular showing distinct optimal dosing patterns.

#### 4.4.4 Combined supplementation strategies

The combination of supplements showed mixed results depending on the protocol. The combined intake of caffeine and L-theanine demonstrated superior improvements in both performance and cognitive function compared to individual supplementation (63). Probiotics combined with vitamin D (4 capsules daily of 2 × 109 CFUs total plus 3,000–4,000 IU vitamin D3) enhanced lactate utilization and anaerobic performance (56). However, combining sodium phosphate with caffeine showed no additional benefits over sodium phosphate alone (43). The effectiveness of dosing strategies appears highly dependent on athlete characteristics, training status, and specific performance demands. The findings emphasize the importance of considering not just the quantity of supplementation but also the timing, duration, and method of administration for optimal performance outcomes. Successful protocols often involve careful consideration of the athlete's competitive schedule, training phase, and individual response to supplementation (31, 53, 60, 62). While some supplement combinations demonstrate synergistic benefits, the inconsistent results across different combinations highlight the complexity of multiple-supplement protocols and the need for careful consideration of interaction effects and individual response patterns in elite athletes.

## 5 Discussion

This systematic review provides a comprehensive analysis of dietary supplement use and efficacy among elite athletes, revealing complex patterns of supplement utilization, varying levels of evidence quality, and important considerations for future research and practice. The findings warrant detailed examination across multiple domains to fully understand their implications for athletic performance and sports nutrition practice. The higher supplement use among elite athletes compared to their non-elite counterparts suggests a performance-driven approach to supplementation. However, this heightened usage does not necessarily correlate with stronger evidence of efficacy. This disconnect between usage patterns and evidence strength indicates that factors beyond proven effectiveness, such as perceived benefits, peer influence, and marketing, may significantly influence supplementation practices in elite sports (104, 105). The variability in evidence quality across different supplements and athletic populations underscores the need for more rigorous research methodologies and standardized approaches to studying supplement efficacy. This systematic review provides comprehensive insights into dietary supplement use among elite athletes, examining 46 studies with 928 participants across various sports. The findings reveal important patterns in supplementation effectiveness, methodological considerations, and future research needs that warrant detailed discussion.

### 5.1 Literature search and study characteristics

The review's extensive search strategy yielded 94 potentially relevant articles, with 46 meeting inclusion criteria after rigorous screening. This relatively small number of eligible studies, despite the widespread use of supplements in elite sports (47.8–93.7% prevalence) reported by Jones (102), highlights the limited high-quality research available on supplement use specifically in elite populations. The gender distribution in the included studies (60% exclusively male participants) reveals a significant bias that aligns with previous observations by Knapik et al. (12), who noted a similar underrepresentation of female athletes in supplementation research. The predominance of endurance sports (particularly cycling) in the research base (28.3% of included studies) suggests an uneven distribution of evidence across sporting disciplines. This finding supports Bishop's (101) observation that supplement effectiveness may be sport-specific, yet research remains concentrated in certain disciplines while others are underrepresented.

### 5.2 Risk of bias and methodological quality

The high PEDro scores (average 10.65 out of 11) among included studies demonstrate strong methodological rigor, contrasting with previous reviews that reported generally poor methodological quality in supplement research. However, the identified limitations in follow-up and intention-to-treat analyses in 9% of studies echo concerns raised by Petróczi et al. (104) regarding the challenges of conducting longitudinal research with elite athletes.

### 5.3 Impact of dietary supplements on performance

The findings regarding caffeine supplementation (3–6 mg/kg body mass) showed (Table 8) consistent benefits for sprint performance and power output, particularly in female athletes. However, individual response variations were significant, supporting findings of (106) which emphasized personalized approaches to supplementation. Beta-alanine supplementation showed varying effectiveness across different sports, with notable improvements in volleyball players' vertical and horizontal jumps (38) but limited benefits in water polo performance (21). This variability in outcomes aligns with (107) findings regarding sport-specific supplement effectiveness. The review revealed interesting findings regarding vitamin D supplementation in professional rugby players, where despite significant increases in serum 25(OH)D concentrations, performance benefits were limited to specific strength measures (33). This selective enhancement supports recent work by Abreu et al. (100) suggesting that nutrient supplementation benefits may be more targeted than previously thought.

**Table 8:** Results on the impact of performance-enhancing dietary supplements on athlete performance.

**Study**	**Sport**	**Supplement**	**Training metrics**	**Physiological markers**
Acar et al. (2024)	Swimming	Caffeine	Significant improvements in 25 m and 50 m freestyle	Not reported
Ávila-Gandía et al. (2021)	Road cycling	β-alanine	Time trial: +3.43% improvement vs−6.20% decline	Higher blood lactate and anion gap
Brisola et al. (2016)	Water Polo	β-alanine	Power output: +30.5% increase; 200 m swim time:−2.2% improvement	Blood lactate decreased
Delleli et al. (2024)	Taekwondo	Caffeine	Improved technical performance metrics	Not reported
Filip-Stachnik et al. (2021)	Judo	Caffeine gum	No significant differences in performance	Not reported
Fye et al. (2021)	Cross-country running	Multi-ingredient	Time to fatigue improved	Higher post-exercise lactate
Garnacho-Castaño et al. (2018)	Cycling	Beetroot juice	No significant time trial differences	Not reported
Graham-Paulson et al. (2016)	Wheelchair rugby	Caffeine	Greater distance covered; Faster sprint times	Higher salivary caffeine
Guo et al. (2024)	Volleyball	Beta-alanine	Improved VO2max and power output	Not reported
Jodra et al. (2020)	Boxing	Caffeine	Peak power improved	Not reported
Jonvik et al. (2018)	Track cycling	Beetroot juice	Time to peak power improved 2.8%	Plasma nitrate/nitrite increased
Karayigit et al. (2021)	Mixed sports	Caffeine	Improved lower-body muscular endurance	Higher lactate values
Lara et al. (2015)	Sprint swimming	Caffeinated energy drink	Power increase of 11%	Blood lactate increased
Liu et al. (2024)	Basketball	Caffeine	Improved power output and accuracy	Not reported
McQuillan et al. (2017)	Cycling	Beetroot juice	No significant performance differences	Not reported
Miraftabi et al. (2021)	Taekwondo	Beetroot juice	No significant performance differences	Not reported
Nyakayiru et al. (2017)	Cycling	Sodium nitrate	No significant time trial differences	Increased plasma nitrate/nitrite
Portillo et al. (2017)	Rugby Sevens	Caffeine	Increased impact frequency	Not reported
Puente et al. (2017)	Basketball	Caffeine	Improved game statistics and jump performance	Not reported
Stadheim et al. (2021)	Cross-country skiing	Caffeine	Increased time to exhaustion	VO2max and lactate increased
Yilmaz et al. (2023)	Curling	Caffeine + L-theanine	Improved shot performance	Improved cognitive performance

All results reported are statistically significant unless otherwise noted.

The analysis of amino acid-based supplements revealed mixed effects, with BCAAs showing improvements in some performance parameters but limited benefits in others (22, 26). This variability in outcomes emphasizes (101)'s argument for more focused approaches to supplement recommendations based on specific performance demands. Sodium bicarbonate supplementation demonstrated complex interactions between dosing protocols and performance outcomes (29, 30, 39). The novel progressive-dose loading protocol (25–100 mg/kg/day) successfully eliminated gastrointestinal side effects while maintaining ergogenic benefits, addressing a key limitation noted in previous research.

### 5.4 Effect of dosing protocols

The review highlighted critical differences between acute and chronic supplementation strategies. Caffeine showed consistent benefits with acute dosing (3-6 mg/kg), while beta-alanine required longer-term protocols for optimal effects. This temporal variation in supplement effectiveness supports (12, 67) emphasis on the importance of appropriate dosing strategies. Gender-specific responses emerged as a significant consideration, particularly in wrestling performance where male athletes showed meaningful improvements with sodium bicarbonate supplementation while female athletes did not. This finding underscores the need for more gender-specific research in sports nutrition, as highlighted by Sousa et al. (106).

## 6 Conclusion

This systematic review of 46 studies encompassing 928 elite athletes provides comprehensive insights into the efficacy of dietary supplements in elite sports performance. The findings reveal a complex landscape where supplement effectiveness varies significantly based on multiple factors including the type of supplement, dosing protocols, timing of administration, and individual athlete characteristics. The high methodological quality of included studies (average PEDro score 10.65/11) provides a robust foundation for evidence-based recommendations, though several important patterns and limitations warrant consideration.

Performance-enhancing supplements demonstrated varying degrees of efficacy across different sporting contexts. Caffeine supplementation (3–6 mg/kg) consistently improved power output and technical performance metrics, particularly in combat sports and team sports, though its effectiveness was notably time-sensitive and context-dependent. Beta-alanine showed sport-specific benefits, with marked improvements in volleyball performance but limited effects in water polo, highlighting the importance of sport-specific supplementation strategies. Nitrate supplementation, despite its popularity, demonstrated limited benefits in elite athletes, suggesting that training status may influence supplement efficacy.

Recovery and adaptation supplements revealed more nuanced outcomes. Amino acid-based supplements, particularly BCAAs combined with arginine and citrulline, showed promise in preventing exercise-induced fatigue, though the translation to performance enhancement varied across studies. Probiotic supplementation, especially when combined with vitamin D, demonstrated benefits in exercise tolerance and recovery, even when direct performance improvements were not observed. These findings suggest that some supplements may contribute to performance enhancement through indirect pathways such as improved recovery and reduced exercise-induced stress.

The review identified critical gaps in current research, particularly the underrepresentation of female athletes (only 10% of studies focused exclusively on female athletes) and the concentration of research in certain sports, especially cycling. These limitations highlight the need for more diverse and representative research populations in future studies. Additionally, the varying protocols and outcome measures across studies make direct comparisons challenging, suggesting a need for more standardized research approaches.

Several key implications emerge for practitioners and athletes. First, the findings strongly support an individualized approach to supplementation, as response variations were significant across different athlete populations. Second, the timing and context of supplement administration proved crucial for maximizing benefits while minimizing potential adverse effects. Third, the effectiveness of progressive loading protocols, particularly for supplements known to cause side effects, suggests that administration strategies should be as carefully considered as the choice of supplement itself.

In conclusion, while dietary supplements can enhance various aspects of elite athletic performance, their effectiveness is highly contingent on multiple factors including proper protocol design, timing, and individual athlete characteristics. The findings emphasize the need for evidence-based, personalized approaches to supplementation in elite sports, moving away from one-size-fits-all recommendations toward more nuanced, context-specific protocols. This review provides a foundation for such approaches while highlighting critical areas for future research to advance our understanding of supplement efficacy in elite athletic performance.

### 6.1 Implications and future directions

This systematic review yields several significant implications for both research and practice in elite sports nutrition. The findings underscore the critical importance of individualized supplementation strategies, moving beyond generic protocols to consider athlete-specific factors such as training status, competition schedule, and physiological response patterns. This personalized approach becomes particularly relevant given the observed variations in supplement effectiveness across different sporting contexts and athlete populations.

The timing and periodization of supplement administration emerge as crucial factors for optimizing performance benefits. The research demonstrates that both acute and chronic supplementation protocols have their place, but their effectiveness depends heavily on the specific supplement and desired outcome. For instance, while caffeine shows consistent benefits with acute administration, other supplements like beta-alanine require structured loading protocols for optimal results. This temporal consideration should inform how supplementation strategies are integrated into training and competition schedules.

Sport-specific considerations have emerged as another critical factor. The varying effectiveness of supplements across different sports suggests that recommendations should be tailored not only to individual athletes but also to specific sporting demands. For example, the differential responses observed between combat sports and endurance activities indicate that supplementation strategies should align with the metabolic and performance demands of each sport.

The findings also highlight the importance of comprehensive monitoring and assessment protocols. Given the variable responses observed across studies, regular evaluation of supplement effectiveness through both objective performance metrics and subjective athlete feedback becomes essential. This monitoring approach should include attention to potential side effects and long-term impacts on athlete health and performance. Moving forward, practitioners should focus on developing integrated supplementation strategies that consider multiple factors including training periodization, competition schedules, and individual athlete characteristics. The implementation of systematic protocols for assessing supplement effectiveness and monitoring athlete responses will be crucial for optimizing supplementation strategies in elite sports. Furthermore, the emerging evidence for supplement combinations suggests the need for careful consideration of potential synergistic effects when designing supplementation protocols.

### 6.2 Limitations

This systematic review encountered several significant limitations that warrant careful consideration when interpreting and applying its findings. The most prominent limitation is the substantial gender imbalance in the research base. With only 10% of studies focusing exclusively on female athletes, our understanding of supplement effectiveness in female elite athletes remains limited. This gender bias restricts the generalizability of findings and highlights a critical gap in our knowledge of how supplementation effects might differ between male and female athletes. The concentration of research in specific sports, particularly cycling (17.4% of studies), creates another significant limitation. This uneven distribution of evidence across sporting disciplines means that some sports are underrepresented in the research base, potentially limiting the applicability of findings to these sports. The varying protocols and outcome measures used across studies present challenges for direct comparisons and meta-analysis, making it difficult to draw definitive conclusions about relative supplement effectiveness.

Methodological limitations also exist despite the generally high quality of included studies. The challenges in maintaining perfect follow-up and completing intention-to-treat analyses in some studies reflect the practical difficulties of conducting research with elite athletes, including competition schedules and injury-related withdrawals. While these limitations affected only a small portion of the included studies, they highlight the ongoing challenges in conducting rigorous research in elite athletic populations. The relatively short duration of many studies represents another limitation, as the long-term effects of supplementation remain poorly understood. Most studies focused on acute or short-term supplementation protocols, leaving questions about the sustained effectiveness and potential long-term impacts of regular supplement use unanswered. Additionally, the varying definitions of “elite” status across studies may impact the comparability of results, though efforts were made to maintain consistent inclusion criteria.

## Data Availability

The original contributions presented in the study are included in the article/Supplementary material, further inquiries can be directed to the corresponding author.
