# Supplementary material for: Efficacy of dietary supplements on sports performance outcomes: a systematic review of evidence in elite athletes
Source: Front Nutr. 2025 Sep 22;12:1675654. doi: 10.3389/fnut.2025.1675654 (PMC12498230; doi:10.3389/fnut.2025.1675654)
Supplement: Supplementary file 1 [file Table_1.pdf]

Appendix

Appendix 1: Search strategy for each database

Database	Search Strategy	Results
PubMed	(("Athletes"[Mesh) OR "elite athlete*"[tiab) OR "professional athlete*"[tiab) OR "olympic athlete*"[tiab) OR "world class athlete*"[tiab) OR "international athlete*"[tiab) OR "high performance athlete*"[tiab) OR "elite sport*"[tiab) OR "professional sport*"[tiab) OR "olympic sport*"[tiab) OR "national team"[tiab)) AND ("Dietary Supplements"[Mesh) OR "Ergogenic Acids"[Mesh) OR "dietary supplement*"[tiab) OR "ergogenic aid*"[tiab) OR "supplemental nutrition"[tiab) OR "nutritional supplement*"[tiab) OR "performance supplement*"[tiab) OR "sports supplement*"[tiab)) AND ("Athletic Performance"[Mesh) OR "Physical Endurance"[Mesh) OR "performance"[tiab) OR "power output"[tiab) OR "endurance performance"[tiab) OR "peak power"[tiab) OR "sport* performance"[tiab) OR "competition performance"[tiab) OR "elite performance"[tiab)) AND ("Randomized Controlled Trial"[Publication Type) OR "Controlled Clinical Trial"[Publication Type) OR "Random Allocation"[Mesh) OR "randomized"[tiab) OR "randomised"[tiab) OR "controlled trial"[tiab) OR "clinical trial"[tiab) OR "RCT"[tiab)) NOT ("Athletic Injuries"[Mesh) OR "Rehabilitation"[Mesh) OR "injury"[tiab) OR "injuries"[tiab) OR "injured"[tiab) OR "recovering"[tiab) OR "recovery"[tiab) OR "rehabilitation"[tiab) OR "return to play"[tiab) OR "return to sport"[tiab) OR "post-injury"[tiab)) AND ("2014/01/01"[Date - Publication) : "2024/12/31"[Date - Publication)) AND english[Language))	78
Scopus	TITLE-ABS-KEY (({elite athlete*} OR {professional athlete*} OR {olympic athlete*} OR {world class athlete*} OR {international athlete*} OR {high performance athlete*} OR {elite sport*} OR {professional sport*} OR {olympic sport*} OR {national team} OR {elite perform*}) AND ({dietary supplement*} OR {ergogenic aid*} OR {supplemental nutrition} OR {nutritional supplement*} OR {performance supplement*} OR {sports supplement*} OR {creatine} OR {caffeine} OR {beta-alanine} OR {protein supplement*} OR {nitrate*} OR {bicarbonate}) AND ({athletic performance} OR {sports performance} OR {power output} OR {endurance performance} OR {peak power} OR {competition performance} OR {elite performance} OR {performance enhancement} OR {strength} OR {speed} OR {aerobic capacity}) AND ({randomized controlled trial} OR {randomised controlled trial} OR {controlled trial} OR {clinical trial} OR {RCT} OR {random allocation} OR {randomized} OR {randomised}) AND NOT ({injury} OR {injuries} OR {injured} OR {rehabilitation} OR {recovering} OR {recovery} OR {return to play} OR {return to sport} OR {post-injury} OR {rehabilitation})) AND DOCTYPE (ar) AND LANGUAGE (english) AND PUBYEAR > 2013 AND PUBYEAR < 2025	4

Database	Search Strategy	Results
Web of Science	TS=((("elite athlete*" OR "professional athlete*" OR "olympic athlete*" OR "world class athlete*" OR "international athlete*" OR "high performance athlete*" OR "elite sport*" OR "professional sport*" OR "olympic sport*" OR "national team" OR "elite player*" OR "world champion*")) AND (("dietary supplement*" OR "ergogenic aid*" OR "supplemental nutrition" OR "nutritional supplement*" OR "performance supplement*" OR "sports supplement*" OR "creatine" OR "protein supplement*" OR "caffeine" OR "beta-alanine" OR "nitrate*" OR "bicarbonate" OR "BCAA*")) AND (("athletic performance" OR "physical performance" OR "power output" OR "endurance performance" OR "peak power" OR "sport* performance" OR "competition performance" OR "elite performance" OR "strength" OR "speed" OR "aerobic capacity" OR "anaerobic capacity")) AND (("randomized controlled trial*" OR "randomised controlled trial*" OR "controlled trial*" OR "clinical trial*" OR "RCT" OR "random allocation") NOT ("injury" OR "injuries" OR "injured" OR "recovering" OR "recovery" OR "rehabilitation" OR "return to play" OR "return to sport" OR "post-injury"))))	3
SPORTDiscus	AB (("elite athlete*" OR "professional athlete*" OR "olympic athlete*" OR "world class athlete*" OR "international athlete*" OR "high performance athlete*" OR "elite sport*" OR "professional sport*" OR "national team" OR "world champion*") AND ("dietary supplement*" OR "ergogenic aid*" OR "nutritional supplement*" OR "performance supplement*" OR "sports supplement*" OR "placebo" OR "creatine" OR "protein supplement*" OR "caffeine" OR "beta-alanine" OR "nitrate*" OR "bicarbonate") AND ("athletic performance" OR "sport* performance" OR "performance enhancement" OR "competition performance" OR "elite performance" OR "power output" OR "endurance performance" OR "peak power" OR "strength" OR "speed" OR "aerobic capacity" OR "anaerobic capacity") AND ("randomized" OR "randomised" OR "controlled trial" OR "clinical trial" OR "RCT" OR "placebo-controlled" OR "double-blind" OR "single-blind") NOT ("injury" OR "injuries" OR "injured" OR "recovering" OR "recovery" OR "rehabilitation" OR "return to play" OR "return to sport" OR "post-injury"))	9
